# Response to clinical evaluation of the effectiveness of fusion‐induced asymmetric transcription assay‐based reverse transcription droplet digital PCR for ALK detection in formalin‐fixed paraffin‐embedded samples from lung cancer

**DOI:** 10.1111/1759-7714.14241

**Published:** 2021-11-20

**Authors:** Cynthia Peñaloza Coronas, Silvia Mariela Montilla Fonseca, Mirna L. Sánchez

**Affiliations:** ^1^ Laboratorio de Farmacología Molecular, Departamento de Ciencia y Tecnología Universidad Nacional de Quilmes‐ CONICET Buenos Aires Argentina; ^2^ Laboratorio de Secuenciación, Dpto. Biología Molecular, Laboratorio de Patología Quirúrgica y Citología de Puebla Puebla Mexico

Dear Editor,

We value the work published by Dr Liu et al.^1^on the design and application of a fusion induced asymmetric transcription assay (FIATA)‐based RT‐*ddPCR* for the detection of ALK rearrangements in non‐small cell lung cancer (NSCLC) samples. It is known that the actual gold standard method for the detection of gene rearrangements are FISH‐based assays, and it is mainly because of the number of fusion variants for each marker, and the fact that it is not necessary for some genes to know the exact fusion product but to prove that the gene is rearranged, in order to provide target therapy that can counter the effects of these rearrangements. Given that the design of the probes is based on the fundamentals of a break‐apart FISH assay, we agree that the protocol proposed in the article will represent an upgrade in contrast to the actual methods for the detection of ALK rearrangements and has the advantage of a quantitative set; thus, providing an invaluable tool in patient monitoring for disease progression.

Based on the principle and design reported in this article, we aimed to design a similar probe for the detection of NTRK rearrangements. Taking into account that all NTRK1, 2 and 3 genes have a tyrosine‐kinase domain that is presumably overexpressed when they are rearranged, by targeting both sides of the breaking point of the NTRK genes, we will be able to identify the rearrangements by an increased number of transcripts of the tyrosine‐kinase domain. Although each fusion of each gene has a different breakpoint, we considered the segment of rupture of the most frequent breakpoints and fusion genes in NSCLC. We are also attempting to design the primer sets of all NTRK genes in an optimized setting, in order to achieve a semi multiplex panel that allows running several PCR fusions assays at the same time. The multiplex setting will lower the total cost of the panel, and it is hoped to be able to translate that to more accessible monitoring tests and follow‐up of oncological patients. To validate this primer set, we will compare the results of our *ddPCR* assay in contrast to IHQ positive samples for these fusions. By contrasting the pattern of the normal tissue with the tumor samples, we expect to see an increased signal of the tyrosine kinase domain of those rearranged samples.

Finally, we again thank Liu et al., for providing such an original design that targets the complexity of the identification of translocations. This finding opens the door to a new way to identify and carry out molecular predictive analysis.
